# Supplementary material for: LimoRhyde2: genomic analysis of biological rhythms based on effect sizes
Source: bioRxiv. 2023 Feb 3:2023.02.02.526897. Preprint. [Version 1] doi: 10.1101/2023.02.02.526897 (PMC9915588; doi:10.1101/2023.02.02.526897)
Supplement: Supplement 2 — Figure S1 LimoRhyde2 moderates amplitude based on standard errors of genes. Scatterplots of difference between raw and posterior peak-to-trough amplitude vs log2 mean standard error of the raw fit for genes in each tissue. Points represent genes. Figure S2 LimoRhyde2’s spline-based model is more flexible and tends to give higher amplitude than the cosinor model. (A) Scatterplots of cosinor posterior peak-to-trough amplitude vs. spline posterior peak-to-trough amplitude for each tissue (indicated at top). Points represent genes. Dashed lines indicate y = x. (B) Time-courses of expression of genes labeled in (A) in the respective tissue (indicated at right). Points represent samples. Curves represent posterior fits for the two models. Figure S3 LimoRhyde2 identifies generally strong rhythms of core clock genes. Posterior peak-to-trough amplitudes and corresponding 90% credible intervals for core clock genes in each tissue. Points represent genes, color represents peak phase for each gene. Dashed lines indicate 0 amplitude. Figure S4 Distributions of rhythmicity based on LimoRhyde2 posterior statistics. Scatterplots of (A) peak-to-trough amplitude vs. mesor and (B) peak-to-trough amplitude vs. peak phase for genes in each tissue (indicated at top). Points represent genes. (C) Histograms of peak phase. All plots include only the top 25% of genes based on amplitude. [file NIHPP2023.02.02.526897v1-supplement-2.pdf]

bioRxiv preprint doi: https://doi.org/10.1101/2023.02.02.526897; this version posted February 3, 2023. The copyright holder for this preprint (which was not certified by peer review) is the author/funder, who has granted bioRxiv a license to display the preprint in perpetuity. It is made available under aCC-BY 4.0 International license.

Figure S1

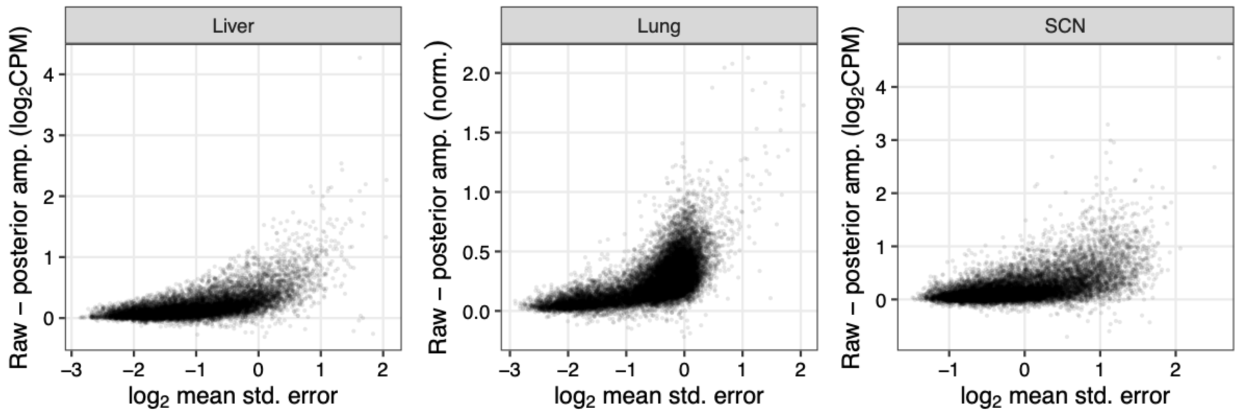

LimoRhyde2 moderates amplitude based on standard errors of genes. Scatterplots of difference between raw and posterior peak-to-trough amplitude vs $\log_2$ mean standard error of the raw fit for genes in each tissue. *Points* represent genes.

bioRxiv preprint doi: https://doi.org/10.1101/2023.02.02.526897; this version posted February 3, 2023. The copyright holder for this preprint (which was not certified by peer review) is the author/funder, who has granted bioRxiv a license to display the preprint in perpetuity. It is made available under aCC-BY 4.0 International license.

Figure S2

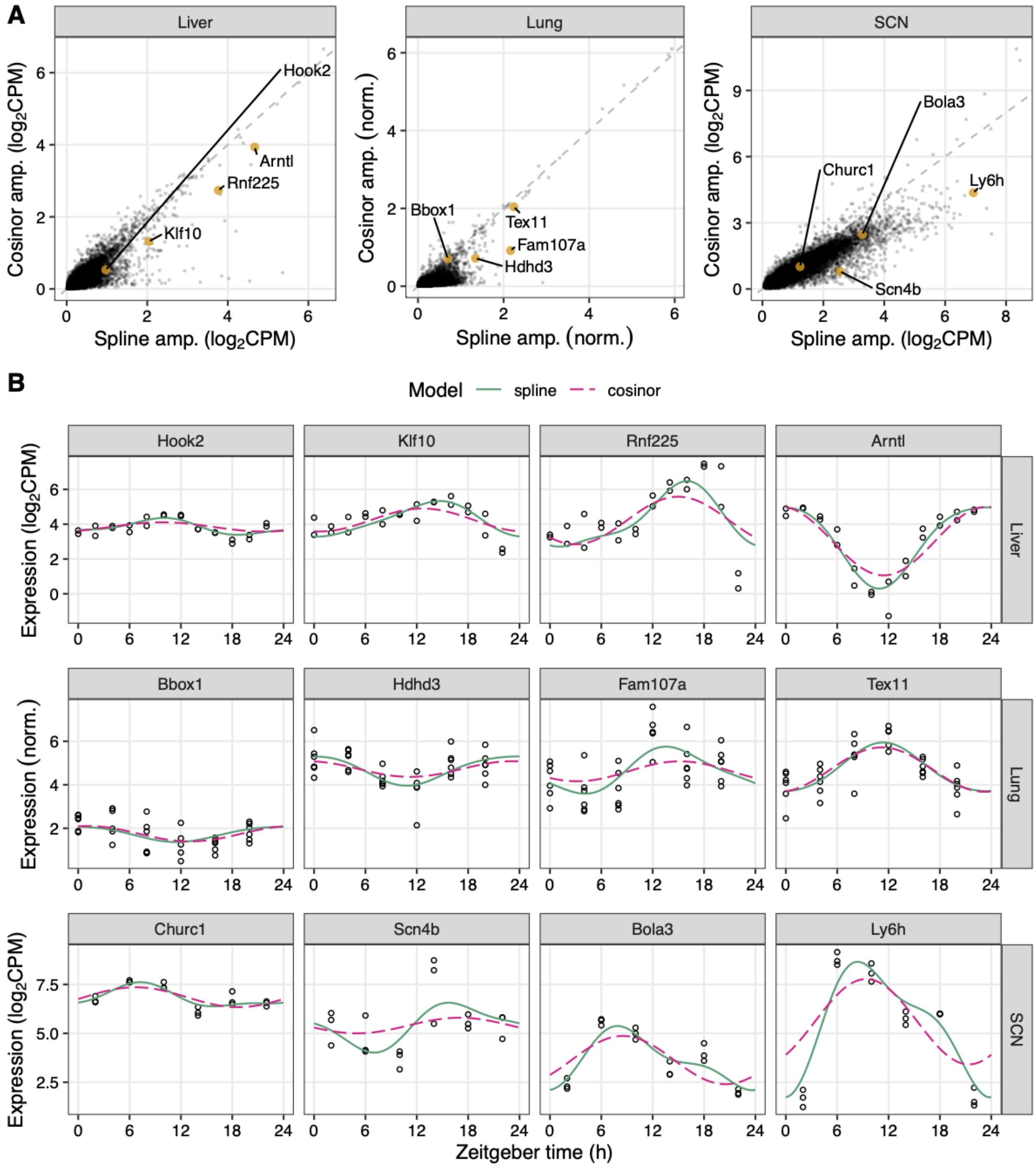

LimoRhyde2's spline-based model is more flexible and tends to give higher amplitude than the cosinor model. (A) Scatterplots of cosinor posterior peak-to-trough amplitude vs. spline posterior peak-to-trough amplitude for each tissue (indicated at *top*). *Points* represent genes. *Dashed lines* indicate y = x. **(B)** Time-courses of expression of genes labeled in (A) in the respective tissue (indicated at *right*). *Points* represent samples. *Curves* represent posterior fits for the two models.

bioRxiv preprint doi: https://doi.org/10.1101/2023.02.02.526897; this version posted February 3, 2023. The copyright holder for this preprint (which was not certified by peer review) is the author/funder, who has granted bioRxiv a license to display the preprint in perpetuity. It is made available under aCC-BY 4.0 International license.

Figure S3

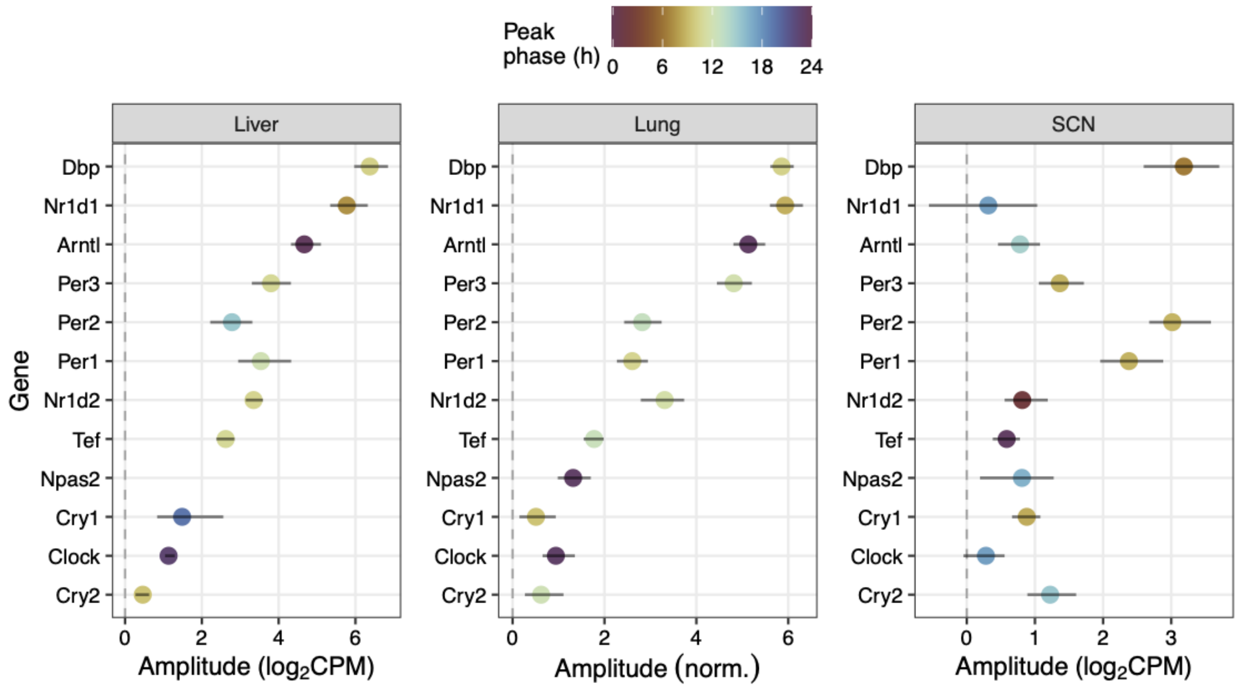

LimoRhyde2 identifies generally strong rhythms of core clock genes. Posterior peak-to-trough amplitudes and corresponding 90% credible intervals for core clock genes in each tissue. *Points* represent genes, *color* represents peak phase for each gene. *Dashed lines* indicate 0 amplitude.

bioRxiv preprint doi: https://doi.org/10.1101/2023.02.02.526897; this version posted February 3, 2023. The copyright holder for this preprint (which was not certified by peer review) is the author/funder, who has granted bioRxiv a license to display the preprint in perpetuity. It is made available under aCC-BY 4.0 International license.

Figure S4

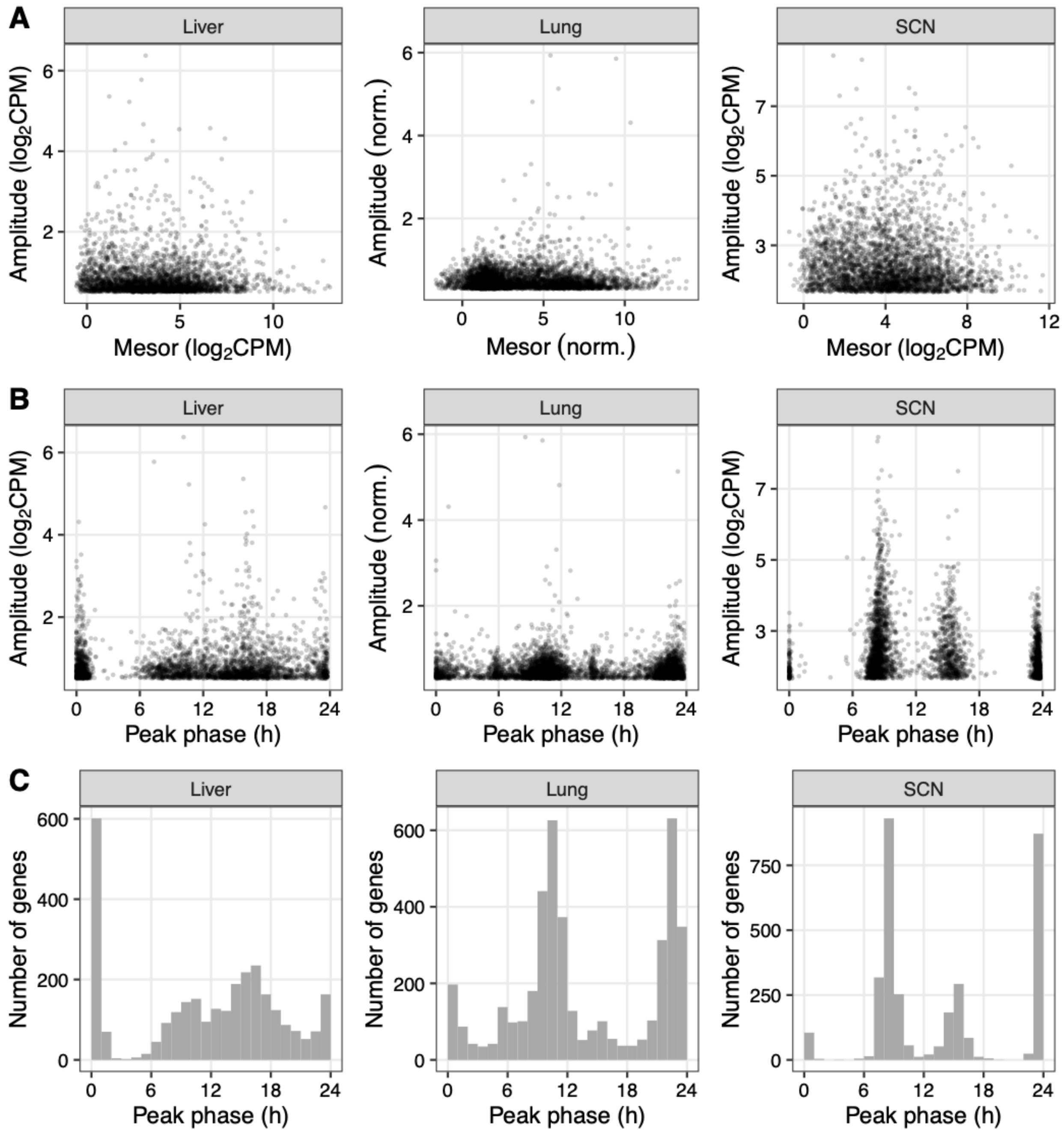

Distributions of rhythmicity based on LimoRhyde2 posterior statistics. Scatterplots of *(A)* peak-to-trough amplitude vs. mesor and **(B)** peak-to-trough amplitude vs. peak phase for genes in each tissue (indicated at *top*). *Points* represent genes. **(C)** Histograms of peak phase. All plots include only the top 25% of genes based on amplitude.
